# IAnimal: a cross-species omics knowledgebase for animals

**DOI:** 10.1093/nar/gkac936

**Published:** 2022-10-27

**Authors:** Yuhua Fu, Hong Liu, Jingwen Dou, Yue Wang, Yong Liao, Xin Huang, Zhenshuang Tang, JingYa Xu, Dong Yin, Shilin Zhu, Yangfan Liu, Xiong Shen, Hengyi Liu, Jiaqi Liu, Xin Yang, Yi Zhang, Yue Xiang, Jingjin Li, Zhuqing Zheng, Yunxia Zhao, Yunlong Ma, Haiyan Wang, Xiaoyong Du, Shengsong Xie, Xuewen Xu, Haohao Zhang, Lilin Yin, Mengjin Zhu, Mei Yu, Xinyun Li, Xiaolei Liu, Shuhong Zhao

**Affiliations:** Key Laboratory of Agricultural Animal Genetics, Breeding and Reproduction, Ministry of Education, Key Laboratory of Swine Genetics and Breeding, Ministry of Agriculture, College of Animal Science and Technology, Huazhong Agricultural University, Wuhan, Hubei 430070, PR China; Frontiers Science Center for Animal Breeding and Sustainable Production, Wuhan, Hubei 430070, PR China; Key Laboratory of Agricultural Animal Genetics, Breeding and Reproduction, Ministry of Education, Key Laboratory of Swine Genetics and Breeding, Ministry of Agriculture, College of Animal Science and Technology, Huazhong Agricultural University, Wuhan, Hubei 430070, PR China; Key Laboratory of Agricultural Animal Genetics, Breeding and Reproduction, Ministry of Education, Key Laboratory of Swine Genetics and Breeding, Ministry of Agriculture, College of Animal Science and Technology, Huazhong Agricultural University, Wuhan, Hubei 430070, PR China; Key Laboratory of Agricultural Animal Genetics, Breeding and Reproduction, Ministry of Education, Key Laboratory of Swine Genetics and Breeding, Ministry of Agriculture, College of Animal Science and Technology, Huazhong Agricultural University, Wuhan, Hubei 430070, PR China; Key Laboratory of Agricultural Animal Genetics, Breeding and Reproduction, Ministry of Education, Key Laboratory of Swine Genetics and Breeding, Ministry of Agriculture, College of Animal Science and Technology, Huazhong Agricultural University, Wuhan, Hubei 430070, PR China; Key Laboratory of Agricultural Animal Genetics, Breeding and Reproduction, Ministry of Education, Key Laboratory of Swine Genetics and Breeding, Ministry of Agriculture, College of Animal Science and Technology, Huazhong Agricultural University, Wuhan, Hubei 430070, PR China; Key Laboratory of Agricultural Animal Genetics, Breeding and Reproduction, Ministry of Education, Key Laboratory of Swine Genetics and Breeding, Ministry of Agriculture, College of Animal Science and Technology, Huazhong Agricultural University, Wuhan, Hubei 430070, PR China; Key Laboratory of Agricultural Animal Genetics, Breeding and Reproduction, Ministry of Education, Key Laboratory of Swine Genetics and Breeding, Ministry of Agriculture, College of Animal Science and Technology, Huazhong Agricultural University, Wuhan, Hubei 430070, PR China; Key Laboratory of Agricultural Animal Genetics, Breeding and Reproduction, Ministry of Education, Key Laboratory of Swine Genetics and Breeding, Ministry of Agriculture, College of Animal Science and Technology, Huazhong Agricultural University, Wuhan, Hubei 430070, PR China; Key Laboratory of Agricultural Animal Genetics, Breeding and Reproduction, Ministry of Education, Key Laboratory of Swine Genetics and Breeding, Ministry of Agriculture, College of Animal Science and Technology, Huazhong Agricultural University, Wuhan, Hubei 430070, PR China; Key Laboratory of Agricultural Animal Genetics, Breeding and Reproduction, Ministry of Education, Key Laboratory of Swine Genetics and Breeding, Ministry of Agriculture, College of Animal Science and Technology, Huazhong Agricultural University, Wuhan, Hubei 430070, PR China; Key Laboratory of Agricultural Animal Genetics, Breeding and Reproduction, Ministry of Education, Key Laboratory of Swine Genetics and Breeding, Ministry of Agriculture, College of Animal Science and Technology, Huazhong Agricultural University, Wuhan, Hubei 430070, PR China; Key Laboratory of Agricultural Animal Genetics, Breeding and Reproduction, Ministry of Education, Key Laboratory of Swine Genetics and Breeding, Ministry of Agriculture, College of Animal Science and Technology, Huazhong Agricultural University, Wuhan, Hubei 430070, PR China; Key Laboratory of Agricultural Animal Genetics, Breeding and Reproduction, Ministry of Education, Key Laboratory of Swine Genetics and Breeding, Ministry of Agriculture, College of Animal Science and Technology, Huazhong Agricultural University, Wuhan, Hubei 430070, PR China; Key Laboratory of Agricultural Animal Genetics, Breeding and Reproduction, Ministry of Education, Key Laboratory of Swine Genetics and Breeding, Ministry of Agriculture, College of Animal Science and Technology, Huazhong Agricultural University, Wuhan, Hubei 430070, PR China; School of Computer Science and Technology, Wuhan University of Technology, Wuhan, Hubei 430070, PR China; Key Laboratory of Agricultural Animal Genetics, Breeding and Reproduction, Ministry of Education, Key Laboratory of Swine Genetics and Breeding, Ministry of Agriculture, College of Animal Science and Technology, Huazhong Agricultural University, Wuhan, Hubei 430070, PR China; Key Laboratory of Agricultural Animal Genetics, Breeding and Reproduction, Ministry of Education, Key Laboratory of Swine Genetics and Breeding, Ministry of Agriculture, College of Animal Science and Technology, Huazhong Agricultural University, Wuhan, Hubei 430070, PR China; Key Laboratory of Agricultural Animal Genetics, Breeding and Reproduction, Ministry of Education, Key Laboratory of Swine Genetics and Breeding, Ministry of Agriculture, College of Animal Science and Technology, Huazhong Agricultural University, Wuhan, Hubei 430070, PR China; Key Laboratory of Agricultural Animal Genetics, Breeding and Reproduction, Ministry of Education, Key Laboratory of Swine Genetics and Breeding, Ministry of Agriculture, College of Animal Science and Technology, Huazhong Agricultural University, Wuhan, Hubei 430070, PR China; Frontiers Science Center for Animal Breeding and Sustainable Production, Wuhan, Hubei 430070, PR China; Key Laboratory of Agricultural Animal Genetics, Breeding and Reproduction, Ministry of Education, Key Laboratory of Swine Genetics and Breeding, Ministry of Agriculture, College of Animal Science and Technology, Huazhong Agricultural University, Wuhan, Hubei 430070, PR China; Frontiers Science Center for Animal Breeding and Sustainable Production, Wuhan, Hubei 430070, PR China; Frontiers Science Center for Animal Breeding and Sustainable Production, Wuhan, Hubei 430070, PR China; Frontiers Science Center for Animal Breeding and Sustainable Production, Wuhan, Hubei 430070, PR China; Key Laboratory of Agricultural Animal Genetics, Breeding and Reproduction, Ministry of Education, Key Laboratory of Swine Genetics and Breeding, Ministry of Agriculture, College of Animal Science and Technology, Huazhong Agricultural University, Wuhan, Hubei 430070, PR China; Frontiers Science Center for Animal Breeding and Sustainable Production, Wuhan, Hubei 430070, PR China; Key Laboratory of Agricultural Animal Genetics, Breeding and Reproduction, Ministry of Education, Key Laboratory of Swine Genetics and Breeding, Ministry of Agriculture, College of Animal Science and Technology, Huazhong Agricultural University, Wuhan, Hubei 430070, PR China; Frontiers Science Center for Animal Breeding and Sustainable Production, Wuhan, Hubei 430070, PR China; School of Computer Science and Technology, Wuhan University of Technology, Wuhan, Hubei 430070, PR China; Key Laboratory of Agricultural Animal Genetics, Breeding and Reproduction, Ministry of Education, Key Laboratory of Swine Genetics and Breeding, Ministry of Agriculture, College of Animal Science and Technology, Huazhong Agricultural University, Wuhan, Hubei 430070, PR China; Frontiers Science Center for Animal Breeding and Sustainable Production, Wuhan, Hubei 430070, PR China; Key Laboratory of Agricultural Animal Genetics, Breeding and Reproduction, Ministry of Education, Key Laboratory of Swine Genetics and Breeding, Ministry of Agriculture, College of Animal Science and Technology, Huazhong Agricultural University, Wuhan, Hubei 430070, PR China; Frontiers Science Center for Animal Breeding and Sustainable Production, Wuhan, Hubei 430070, PR China; Key Laboratory of Agricultural Animal Genetics, Breeding and Reproduction, Ministry of Education, Key Laboratory of Swine Genetics and Breeding, Ministry of Agriculture, College of Animal Science and Technology, Huazhong Agricultural University, Wuhan, Hubei 430070, PR China; Frontiers Science Center for Animal Breeding and Sustainable Production, Wuhan, Hubei 430070, PR China; Key Laboratory of Agricultural Animal Genetics, Breeding and Reproduction, Ministry of Education, Key Laboratory of Swine Genetics and Breeding, Ministry of Agriculture, College of Animal Science and Technology, Huazhong Agricultural University, Wuhan, Hubei 430070, PR China; Frontiers Science Center for Animal Breeding and Sustainable Production, Wuhan, Hubei 430070, PR China; Key Laboratory of Agricultural Animal Genetics, Breeding and Reproduction, Ministry of Education, Key Laboratory of Swine Genetics and Breeding, Ministry of Agriculture, College of Animal Science and Technology, Huazhong Agricultural University, Wuhan, Hubei 430070, PR China; Frontiers Science Center for Animal Breeding and Sustainable Production, Wuhan, Hubei 430070, PR China; Hubei Hongshan Laboratory, Wuhan, Hubei 430070, PR China; Key Laboratory of Agricultural Animal Genetics, Breeding and Reproduction, Ministry of Education, Key Laboratory of Swine Genetics and Breeding, Ministry of Agriculture, College of Animal Science and Technology, Huazhong Agricultural University, Wuhan, Hubei 430070, PR China; Frontiers Science Center for Animal Breeding and Sustainable Production, Wuhan, Hubei 430070, PR China; Hubei Hongshan Laboratory, Wuhan, Hubei 430070, PR China

## Abstract

With the exponential growth of multi-omics data, its integration and utilization have brought unprecedented opportunities for the interpretation of gene regulation mechanisms and the comprehensive analyses of biological systems. IAnimal (https://ianimal.pro/), a cross-species, multi-omics knowledgebase, was developed to improve the utilization of massive public data and simplify the integration of multi-omics information to mine the genetic mechanisms of objective traits. Currently, IAnimal provides 61 191 individual omics data of genome (WGS), transcriptome (RNA-Seq), epigenome (ChIP-Seq, ATAC-Seq) and genome annotation information for 21 species, such as mice, pigs, cattle, chickens, and macaques. The scale of its total clean data has reached 846.46 TB. To better understand the biological significance of omics information, a deep learning model for IAnimal was built based on BioBERT and AutoNER to mine ‘gene’ and ‘trait’ entities from 2 794 237 abstracts, which has practical significance for comprehending how each omics layer regulates genes to affect traits. By means of user-friendly web interfaces, flexible data application programming interfaces, and abundant functional modules, IAnimal enables users to easily query, mine, and visualize characteristics in various omics, and to infer how genes play biological roles under the influence of various omics layers.

## INTRODUCTION

With the rapid development of high-throughput sequencing technology, the quantity of data in omics layers has increased dramatically. The integration analysis of multi-omics data has brought unprecedented opportunities for the interpretation of gene regulation mechanisms and the comprehensive analysis of biological systems (1). For example, the Encyclopedia of DNA Elements (ENCODE) project aims to precisely and comprehensively delineate the segments that encode functional elements in the human and mouse genomes using large amounts of multi-omics data, which include genome, transcriptome and epigenome data; 926 535 human candidate cis-regulatory elements (cCREs) and 339 815 mouse cCREs have been identified so far (2). The Functional Annotation of ANimal Genomes (FAANG) project is working to decipher the function of genome segments with multi-omics data, and to date it has completed the analysis of 14 animals, including pigs, cattle, and salmon (3). However, several key challenges have emerged in the development and utilization of multi-omics data. First, various types of complicated data sources and different descriptive standards of data notably increase the difficulty of data collection and cleaning. Second, the huge amount of omics data requires efficient methods of data analysis, storage, and retrieval. Finally, intelligent methods need to be developed to integrate, mine, and interpret various types of omics data.

Compared with model animals, like mice, multi-omics integration research progress for livestock animals (e.g. pigs), companion animals (e.g. cats), and wild animals (e.g. pandas) lags far behind. One of the main reasons is that the data volume of these animal species is relatively small, at ∼0.2–5% that of mice (Supplementary Figure S1). Additionally, the publicly available omics data for these animal species are not well standardized because the data come from different projects, and there is a lack of unified methods for systematic collation of basic sample information, quality control, and analysis, which makes these data difficult to reuse.

There is evidence that reusing publicly available omics data facilitates new biological discoveries. For example, in our previous study (4), almost all publicly available microRNA (miRNA) data for pigs were collected, cleaned, and analyzed, which tripled the annotation number of pig miRNAs, and this also improved the integrity of annotation information for half of the known miRNAs. Therefore, many studies have tried to clean and normalize the highly heterogeneous omics data of animals. For example, Genome Variation Map (GVM) mainly focuses on genome variation (5), Ruminant Genome Database (RGD) focuses on ruminant gene functional research (6), and The Animal QTL Database (AnimalQTLdb) provides abundant quantitative trait loci (QTL) information of animals (7). However, existing databases focus mainly on a single type of omics, but multi-omics data that include DNA, RNA and proteins are necessary to reveal causal relationships between genes and traits from a holistic perspective of biological systems. Meanwhile, applying massive multi-omics data of model animals like mice to build an integrative multi-omics model to adapt to other animal species is expected to break the bottleneck caused by insufficient data. The multi-omics data of multiple species can in turn be used to refine the study of gene function in model animals. Therefore, it is important to develop a platform for the comprehensive collection of multi-omics data on various animal species and the facilitation of cross-species omics research.

At present, most animal omics databases follow a strategy wherein omics data are analyzed in advance, and fixed conclusions are provided to users. This strategy is very effective for solving specific problems, but it sacrifices the flexibility and reusability of data, and it indirectly wastes computing resources and time. For example, in Animal-eRNAdb (8), enhancer RNA (eRNA) expression level can be queried easily in all available individuals for a given species, but the data need to be re-downloaded, cleaned, and analyzed if the expression level in a particular subset of individuals needs to be fetched. Similarly, GVM (5) allows users to query genotype information easily on any particular marker site of all available individuals in a given species, but it does not support queries or computational operations at the individual level. More flexible databases are required to offer omics data processing at the individual level, which can significantly promote the reusability and mining efficiency of omics data.

The integrated analysis of multi-omics data is a difficult problem to solve. The commonly used method is data stacking, which is relatively simple to implement but has high false positive and false negative rates that can be effectively reduced by designing appropriate statistical models for specific data sets in the case of ignoring the limitation of specific scenarios and experiments. With the arrival of the third development wave of artificial intelligence, deep learning has become one of the most promising research methods for multi-omics integration due to its good compatibility with heterogeneous data and its powerful big data processing capabilities (9). In one study, a convolutional neural network (CNN) model was utilized to integrate the information of genome, transcriptome, and quantitative trait loci/gene/nucleotide (QTX) in pigs and to provide a score to assess the causal relationship of each ‘gene-trait’ pair (10). Compared with single omics, the CNN model trained by multi-omics data improved the mining efficiency of key genes underlying specific traits, but the limited multi-omics data for pigs also posed an obstacle to the further improvement of scoring accuracy. Making full use of massive, cross-species multi-omics data through transfer learning is expected to solve this problem in some animal species with insufficient multi-omics data. In addition, the rapid development of natural language processing technology makes it possible to efficiently mine gene-trait relationships in a large quantity of literature, thus helping to predict gene functions and to improve the interpretability of results from integrated analysis of multi-omics data.

In this study, we constructed IAnimal, which is an individual-level, cross-species, multi-omics knowledgebase. It includes individual level omics data for genome (WGS, whole genome sequencing), transcriptome (RNA-Seq, RNA sequencing), and epigenome (ChIP-Seq, chromatin immunoprecipitation with high-throughput sequencing, and ATAC-Seq, assay for transposase-accessible chromatin with high-throughput sequencing) data for 21 animal species, including mice, pigs, cattle, chickens and macaques. In addition, IAnimal also contains a large quantity of literature abstracts to reveal how each omics affects the traits through genes. Unified standards were used to clean, analyze, and structure these omics data based on engineering approaches and crowdsourcing ideas. Data-application programming interfaces (APIs) were also developed at the individual level to settle upon a convenient approach for the use of structured data.

## DATA COLLECTION AND PROCESSING

### Data collection

Genome data, high-throughput omics data, and information extracted from the literature of 21 animal species (including mice, pigs, cattle, chickens and macaques) were collected to construct a cross-species, multi-omics, knowledgebase. Because the quantity of data for mice far exceeds those of other species, a certain number of representative samples were selected by excluding highly similar ones. In contrast, data for other species were collected as comprehensively as possible. Genome sequences and annotations of all species were obtained from the Ensembl database (11), high-throughput sequencing reads were downloaded from the SRA (12) and EBI (13) databases, and literature abstracts were acquired from the NCBI database (14) through the Entrez interface. After quality control on these data, the final information used in IAnimal included 2 794 237 literature abstracts and 61,191 individual level omics data from WGS, RNA-Seq, ChIP-Seq and ATAC-Seq and genome annotation information for 21 species. The scale of clean data was approximately 846.46 TB (Table 1).

**Table 1.** Data summary of the IAnimal knowledgebase

Species	WGS	RNA	ChIP	ATAC	Literature	Project	Tissue	Variation(M)	Bases(TB)
*Ailuropoda melanoleuca*	58	133	0	0	2534	18	21	12.42	2.49
*Anas platyrhynchos*	1162	819	0	4	1408	87	30	44.27	18.38
*Anser cygnoides*	283	134	0	8	130	18	10	22.67	5.31
*Balaenoptera musculus*	1	2	0	0	629	3	3	6.17	0.13
*Bos taurus*	983	3995	216	158	291 242	243	85	52.71	76.89
*Camelus dromedarius*	38	28	0	0	4197	12	10	10.17	1.7
*Canis lupus familiaris*	2116	2581	95	9	225 467	263	126	47.57	134.04
*Capra hircus*	961	1355	0	5	1015	159	60	65.66	64.24
*Equus asinus*	189	61	8	0	53 280	13	14	16	2.66
*Equus caballus*	538	2192	135	18	58 089	155	95	35.71	42.64
*Felis catus*	311	180	0	0	92 331	44	46	79.49	25.91
*Gallus gallus*	1108	4098	533	161	108 208	462	111	37.16	53.26
*Loxodonta africana*	11	23	0	0	567	9	6	14.3	1.55
*Macaca mulatta*	696	7318	222	149	37 963	246	127	107.18	129.66
*Mus musculus*	80	8983	2499	544	1 644 283	1340	132	16.47	77.99
*Oryctolagus cuniculus*	49	1424	67	12	234 595	91	45	106.05	12.33
*Ovis aries*	877	2682	90	8	8715	222	75	71.91	61.83
*Panthera leo*	41	2	0	0	5439	5	6	13.93	1.14
*Panthera tigris*	8	2	0	0	914	4	4	12.11	0.99
*Sus scrofa*	1311	8626	647	130	23 092	652	218	95.2	132.92
*Ursus thibetanus*	14	0	0	0	139	2	1	10.44	0.4
**ALL**	**10 853**	**44 638**	**4512**	**1206**	**2 794 237**	**4030**	**256**	**877.6**	**846.46**

How to efficiently collect, clean, analyze and store large omics data from widely distributed sources, different data formats and uneven quality is always a great challenge. Considering the various characteristics of omics data, unified standards and platforms were designed in this study. First, an automatic download, analysis, and storage system for omics data was developed using technologies such as Docker, Nextflow (15) and PostgreSQL. Then, for high-throughput data that needed manual cleaning, an NGS cleaning program (Supplementary Figure S2) based on the idea of ‘crowdsourcing’ was established, in which volunteers viewed and processed the data simultaneously, and potential errors were corrected by mutual verification. Meanwhile, the Label Studio (16) platform (https://github.com/heartexlabs/label-studio) was employed to conduct online labeling for the literature.

### Functional annotation of animal genomes

The loci, sequences, structures, and other basic information of genes for all species were parsed from genome and annotation files that were downloaded from the Ensembl (Release 104) database (11). To unify the functional annotation standards and provide convenient exploration, InterProScan (V5.27) (17) and KofamScan (V1.3.0) (18) were utilized with Swiss-Prot (19), Kyoto Encyclopedia of Genes and Genomes (KEGG) (20), Gene Ontology (GO) (21), Pfam (22), InterPro (23) and KOG (24) databases to obtain the functions of 570,628 genes in all species. The percentages of genes that had annotation information from Swiss-Prot, KEGG, GO, Pfam, InterPro, and KOG were 70.71%, 55.10%, 51.35%, 66.15%, 68.37% and 63.68%, respectively (Supplementary Table S1).

### Gene family and core gene set analysis

To facilitate the cross-species comparison of genes, the longest coding transcript was retained for isoforms, and OrthoFinder (V2.5.4) (25) was applied to group them into 30 206 clusters. Consistent with a previous study (26), we also defined core gene sets that are common to all species at a certain phylogenetic level and potentially dispensable gene sets that show presence/absence variations across species at the same phylogenetic level. According to the distribution of genes for each species in these clusters, the core and dispensable gene families were counted in different evolutionary branches, and core genes of different species were identified simultaneously at different phylogenetic levels, which included phylum, class, order, family, genus, and species (Supplementary Figure S3B).

### Processing of WGS-seq data

All collected WGS data were processed using standard bioinformatics pipelines. By using SRAToolkit (V2.8.2) (27), raw data were first converted to fastq files, which were subsequently trimmed by removing adapters and low-quality (‘-W 4 -M 20 -q 20 -u 40 -n 5 -l 15’) bases using fastp (V0.12.4) (28). The remaining high-quality reads were aligned against the reference sequence by using BWA (V0.7.17) (29). Uniquely mapped reads were used for detection of short variants with Sentieon (V202010.02) (30). To obtain highly confident short variants, samples with sequencing depth <3 and coverage <70% were removed. GATK (V4.0.3.0) (31) was next employed using the parameter ‘QUAL < 30.0 | | QD < 2.0 | | FS > 60.0 | | MQ < 40.0 | | SOR > 3.0 | | ReadPosRankSum < −8.0’ / ‘QUAL < 30.0 | | QD < 2.0 | | FS > 200.0 | | SOR > 10.0 | | ReadPosRankSum < −20.0 | | MQ < 40.0 | | MQRankSum < −12.5’ to retain high-quality, short variants that were then annotated by ANNOVAR (V2018Apr16) (32). Finally, IAnimal recorded 877 598 274 variations in 10 835 WGS samples from 21 species; approximately 35% of the variations were mapped to dbSNP (V155) (33), which improved usability and comparability. Because variations can reflect genetic distance, FastTree (V2.1.10) (34) was used to construct a phylogenetic tree of all samples, which has been embedded in the Population module.

### Processing of RNA-seq data

Like WGS data, all collected RNA-seq datasets were also processed through a standard bioinformatics pipeline. After conversion and trimming, the remaining high-quality reads were aligned against the reference sequence by HISAT2 (V2.2.1) (35), and then alignments were fed to StringTie (V2.1.7) (36) to assemble the transcripts and to quantify the expression levels of all genes. To ensure the accuracy of quantification, samples with aligned reads >6 million were retained. To prevent interference from abnormal samples, the median value was applied to represent the gene expression in the heatmap, and outliers were deleted by using the method of Tukey's fences in the boxplot. The specific formula was as follows:

$$\begin{equation*}\left[ {Q1 - k*\left( {Q3 - Q1} \right),Q3 + k*\left( {Q3 - Q1} \right)} \right],\end{equation*}$$

where $Q1$ and $Q3$ represent the first and third quartiles of Euclidean distance observations, respectively, and $k$ is a nonnegative constant, where $k\ = {\rm{\ }}1.5$ or $k\ = {\rm{\ }}3$ indicates an ‘outlier,’ and $k$ was set to 3 in this study. At last, the Pearson correlation coefficient between two genes without considering tissue type, breed, or developmental stage in a species was defined as gene co-expression coefficient (GCC).

### Processing of ChIP-seq and ATAC-seq data

All collected ChIP-Seq and ATAC-Seq datasets were first required to pass conversion and quality control with both fastp (V0.12.4) (28) and Chromap (V0.2.3) (37). MACS3 (V3.0.0a7) (38) was used to call peaks with the parameter ‘-p 0.01 –nomodel –shift -75 –extsize 150 –keep-dup all -B –SPMR’ for ATAC-Seq data and ‘-q 0.01 –nomodel –shift 0 –extsize $x –keep-dup all -B –SPMR’ for ChIP-Seq data, where $x was calculated by SPP (V2.0.1) (39). The bedGraph files generated above were converted to BigWig format by bedGraphToBigWig (V2.9) (40) for downstream analysis and visualization in JBrowser. To facilitate the comparison of enrichment signals in the specified region of different samples, the genome was divided into bins with a length of 200 bp in which the enrichment signals were counted by bigWigAverageOverBed (V2.0) (40). It is worth noting that because the amount of ChIP-Seq and ATAC-Seq data for the vast majority of species was much smaller than that of RNA-Seq and WGS data, a relatively loose filtering criterion was established, namely that only samples with <2000 peaks were deleted. Users can flexibly select interesting samples for mining and visualization through the interface provided by IAnimal.

### Processing of literature data

The BioBERT (41) and AutoNER (42) models were built (Supplementary Results) to process the literature data, and the accuracy, precision, recall, and F1-Measure of the optimized model were 89.95%, 78.39%, 32.19% and 45.64%, respectively. For BioBERT, the manual labeling of gene and phenotype entities in 1760 abstracts was performed in the Label Studio platform (16), and a fine-tuned BioBERT model was built using transfer learning. For AutoNER, gene dictionaries were constructed with gene IDs, names, and descriptions of all species, and phenotype dictionaries were constructed with terms from Mammalian Phenotype Ontology (43) and Vertebrate Trait Ontology (44). Based on these two models, the gene and phenotype entities were identified in all literature abstracts, and the union was obtained. To offer convenience for exploring the relationships between genes and traits, gene entities were mapped to both gene ID and gene name, and only the sentences that contained both genes and traits were kept for query, feedback, and visualization.

## SYSTEM DESIGN AND IMPLEMENTATION

IAnimal is a decomposing system primarily based on the Vue front-end and SpringBoot back-end framework. To facilitate the storage and invocation of big omics data, we used MySQL and MongoDB as storing systems, and MyBatis and Redis as persistent layers. To use third-party programs conveniently, IAnimal uses docker package software for back-end services such as JBrowse2 (V1.7.10) (45), SequenceServer (V2.0.0) (46), and Primer3web (V0.4.0) (47). IAnimal is freely available to the public, accessible on both computers and mobile devices without login or registration, and it has been optimized for multiple browsers, including Chrome (recommended), Internet Explorer, Opera, Firefox, Microsoft Edge and Safari.

## DATABASE CONTENT AND USAGE

### Overview of IAnimal

**Figure 1.**
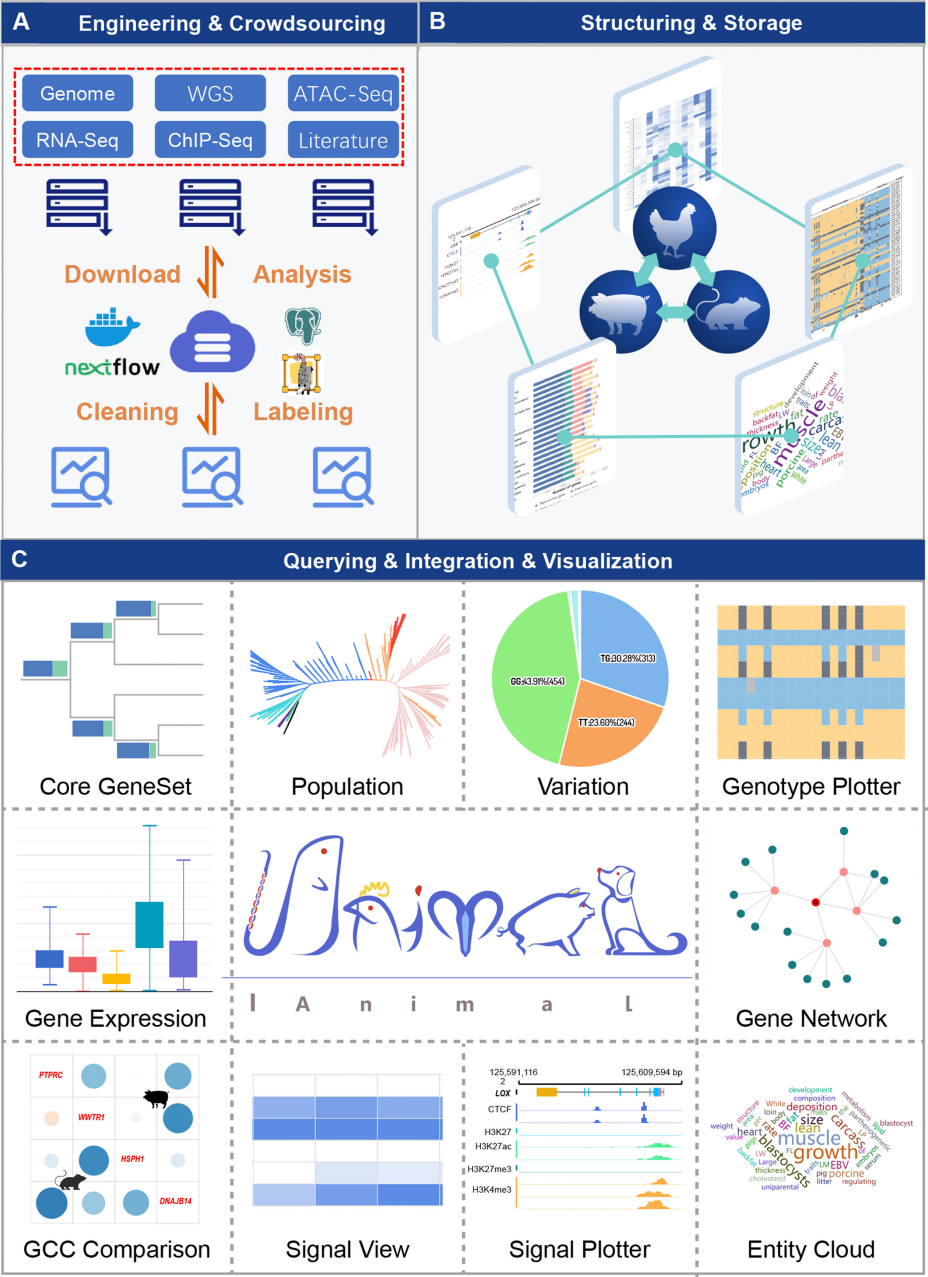
Schematic diagram of IAnimal. (**A**) Pipelines of data pre-processing. (**B**) Back-end data of various omics information stored in IAnimal. (**C**) Gallery of functional modules in IAnimal.

IAnimal is committed to helping users excavate gene functions by using big, cross-species, multi-omics data, which can make full use of massive public data and, simultaneously, reduce the energy consumption caused by tremendously repetitive calculations. Based on engineering and crowdsourcing concepts, IAnimal completes data collection and analysis efficiently (Figure 1A, B), develops flexible data APIs to facilitate data invocation and excavation, and provides user-friendly functional modules to make the knowledgebase easy to use (Figure 1C). The current implementations of the IAnimal knowledgebase contain 25 modules in five core sections (Genome, Transcriptome, Epigenome, Literature and Tools) and three additional auxiliary sections (Taxonomy, Download and Help). The core sections are mainly developed for the purpose of convenient data query, excavation, and visualization, and the auxiliary sections help users obtain additional information and documents provided by the knowledgebase. Users can browse and preview the functions of candidate genes rapidly through the gene search module located on the homepage. This module integrates various omics information of genes to help users explore their potential functions, then users can jump to the relevant omics section to explore the functions of each gene at a specific omics level and, finally, a series of relevant toolsets can be applied to the downstream excavating analysis of gene functions.

### Gene Search module with integrated multi-omics information

**Figure 2.**
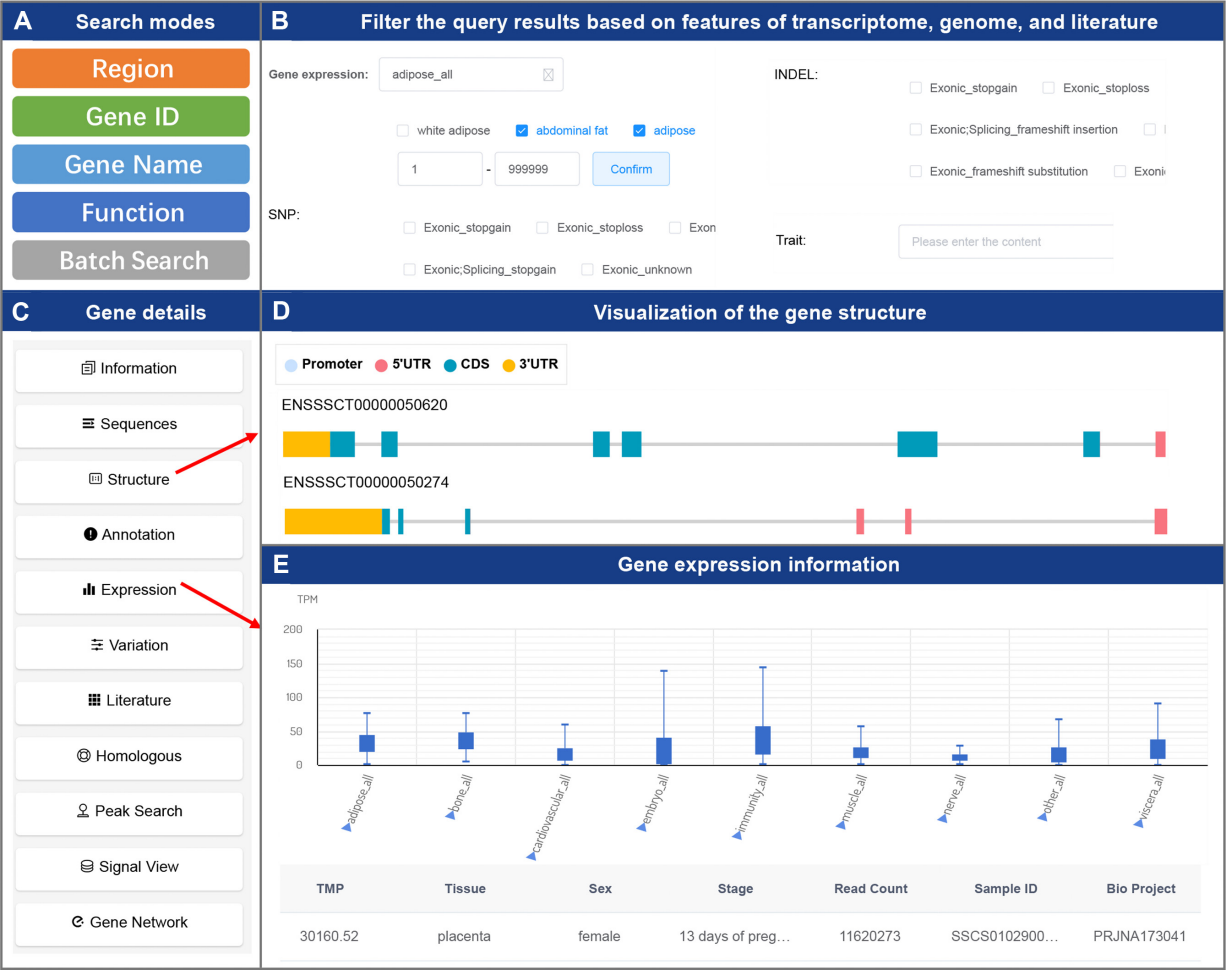
Interface of the Gene Search module. (**A**) There are five different search modes in the Gene Search module. Users can select an appropriate mode to obtain the gene sets of interests. (**B**) Users can filter the query results based on features of transcriptome, genome, and literature. When there are too many query results, users can narrow down the candidate gene sets by this mode. (**C**) Gene information at different omics layers. This mode integrates 11 kinds of information for a queried gene, so users can infer the gene's potential biological functions quickly. (**D**) Visualization of the gene structure. Users can view the structure information of different gene transcripts intuitively. (**E**) Gene expression information. This includes the gene expression of each individual and the gene expression in different tissues.

A quick way to utilize multi-omics information is to search the genes of interest through the Gene Search module located on the homepage, which supports searching by gene name, gene id, genomic region or functional annotation (Figure 2A). Through the advanced search function, users can perform more flexible gene searches, which include batch search and screening of large-scale genes. When there are many search results, users can filter the search results by gene expression level in the specified tissue, the type of mutation contained in the gene, or the gene function given by the literature group (Figure 2B). The results integrate ‘basic information’, ‘sequence’, ‘structure’ (Figure 2D), ‘functional annotation’, ‘expression levels’ (Figure 2E), ‘variant’, ‘literature entities’, ‘homologous genes’, ‘peak signal’ and ‘gene network’ for all genes, and users can infer the potential biological functions of the genes quickly from this information (Figure 2C). Here, the omics information of genes is integrated mainly by using the default parameters, where users can explore the functions of candidate genes through specific modules and data APIs in IAnimal.

### Genome section

The Genome section contains six modules: Gene Annotation, Gene Family, Core GeneSet, Genome Information, Variation and Population. The Gene Annotation module is used mainly to help users query the annotation of a specified gene in databases such as Swiss-Prot, KEGG, GO, Pfam and InterPro. The Gene Family module is designed to query genes and gene families, to explore gene functions from the gene family level, and then to realize the comparison of gene functions within and between species (Supplementary Figure S3A). The Core GeneSet module provides conserved/dispensable gene families in different evolutionary branches and conserved genes of all species at different phylogenetic levels (e.g. phylum/class/order/family/genus/species, Supplementary Figure S3B). Users can download relevant information by interacting with the visual images. The Genome Information module provides basic information on the genome in IAnimal, which is convenient for obtaining the same genome for downstream analysis.

**Figure 3.**
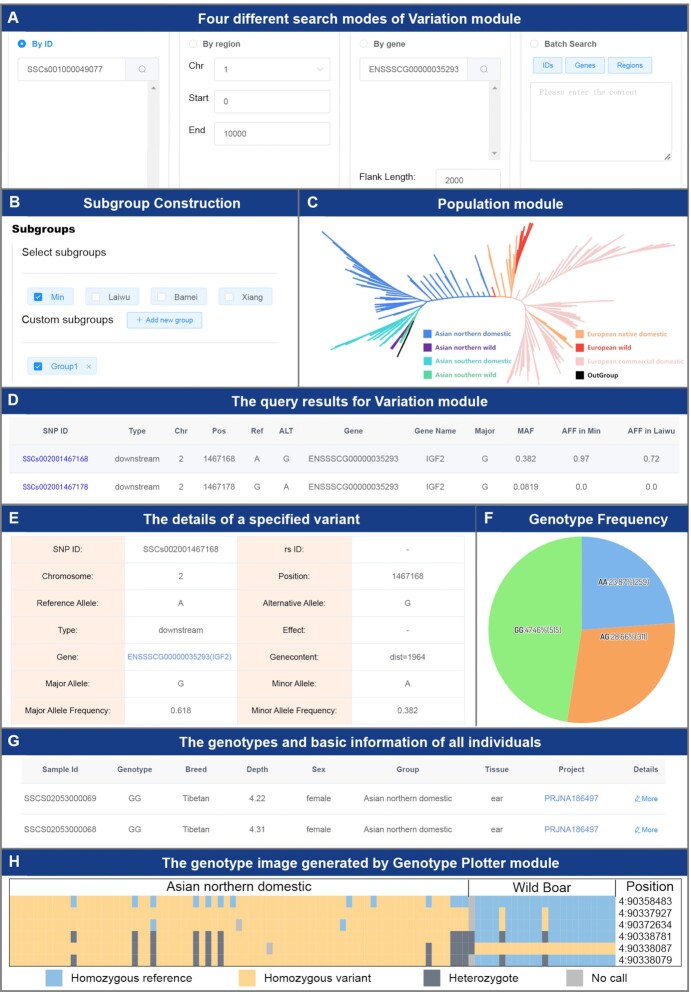
The main functions and usage of the Genome section. (**A**) There are four different search modes in the Variation module. Users can select an appropriate mode to search the variations of interest. (**B**) There are two methods of constructing subgroups. Subgroups can be constructed through breed information or sample IDs. (**C**) The Population module helps users customize subgroups. Through the sample information and phylogenetic tree of this module, users can better understand the population structure and construct subgroups. (**D**) An example of Variation module search results. The similarities or differences of gene frequencies among subgroups can be easily compared. (**E**) The details of a specified variant. (**F**) The genotype frequency of a specified locus. (**G**) The genotypes and basic information of all individuals. Users can select individuals of interest for downstream analysis. (**H**) The genotype image generated by the Genotype Plotter module. The genotype alleles of homozygous reference, homozygous variant, heterozygote, and missing (no call) are marked in blue, yellow, dark grey and light grey, respectively.

The Variation module is the most important function in this section. With the aid of this module, users can retrieve interesting variant loci in the form of variant ID, gene ID/Name and genome region (Figure 3A), and users can also construct one or more interesting subpopulations through breed information or sample ID (Figure 3B). To make full use of individual information to construct subpopulations, this section also provides a Population module to help users understand the basic information and evolutionary relationships of samples (Figure 3C). The Variation module will calculate gene frequencies for all specified subpopulations, so users can quickly compare the similarities and differences of variant loci among these subpopulations (Figure 3D). Users can further filter the variant loci of interest based on the comparison results among these subpopulations and obtain detailed annotation information for these variant loci and their distribution in all samples (Figure 3E, F). Furthermore, the genotype data of all individuals can be obtained through the download interface provided by this module to achieve more flexible downstream analysis and exploration (Figure 3E). In addition, to facilitate users to visualize the genotype of specified samples, IAnimal also provides the Genotype Plotter module based on our flexible data API (Figure 3H). Users only need to input the variant ID and sample ID of interest, and the module will output the high-quality genotype image, which can be used for publication directly.

### Transcriptome section

**Figure 4.**
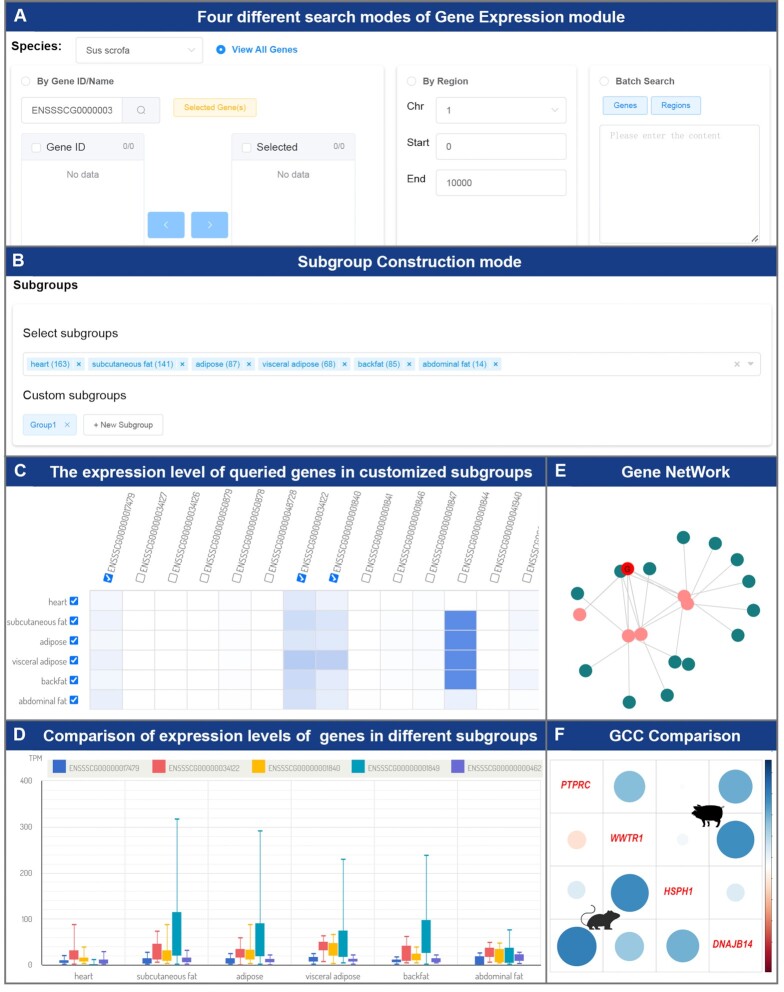
The main functions and usage of the Transcriptome section. (**A**) There are four different search modes in the Gene Expression module. Users can select an appropriate mode to search the gene sets of interest. (**B**) There are two methods of constructing subgroups. Users can construct subgroups through tissue information or sample IDs. (**C**) The expression levels of queried genes in customized subgroups. Users can view the expression levels of genes in different subgroups conveniently. (**D**) Comparison of expression levels of specified genes in different subgroups. Users can select genes and subgroups of interest from a heatmap to be displayed in a boxplot for comparison. (**E**) The regulatory network of the specified gene and other genes. Users can obtain and visualize the gene set associated with the specified gene through the gene ID and the GCC threshold. (**F**) Visualization of the regulation patterns among *PTPRC*, *WWTR1*, *HSPH1* and *DNAJB14* between pigs and mice by using the GCC Comparison module.

The Transcriptome section contains three modules: Gene Expression, Gene Network, and GCC Comparison. Users can retrieve the expression level of the gene of interest in different samples through gene ID/Name or genome region, and batch search is also available for multiple genes (Figure 4A). Since the sample size of the transcriptome is generally large, users often expect to compare the expression levels of genes across several specific subgroups. Therefore, this module provides two modes (custom grouping and quick grouping by tissue) to help users generate subgroups of interest rapidly (Figure 4B). Finally, the expression levels of genes in each subgroup are displayed in a heatmap (Figure 4C), and users can select the genes and subgroups of interest from the heatmap to be displayed in a boxplot for comparison (Figure 4D).

The Gene Network module in this section can also construct a GCC matrix for all genes. Users can obtain and visualize the gene set (target genes) related to the specified gene (query gene) and indirect genes related to the target genes (Figure 4E) through the gene ID and the GCC threshold (the default setting is that the absolute value of the GCC is >0.5). By default, only the top 10 genes in the absolute value of GCC are displayed, and the user can increase or decrease the number of genes to be displayed by changing the corresponding parameters. To compare the differences in the regulation patterns of genes in different species (Figure 4F), this section also provides a GCC Comparison module to obtain the GCC of a specified gene set in two different species. Users only need to select two species and enter a gene set to visually compare the GCC among the gene sets between the two species.

### Epigenome section

**Figure 5.**
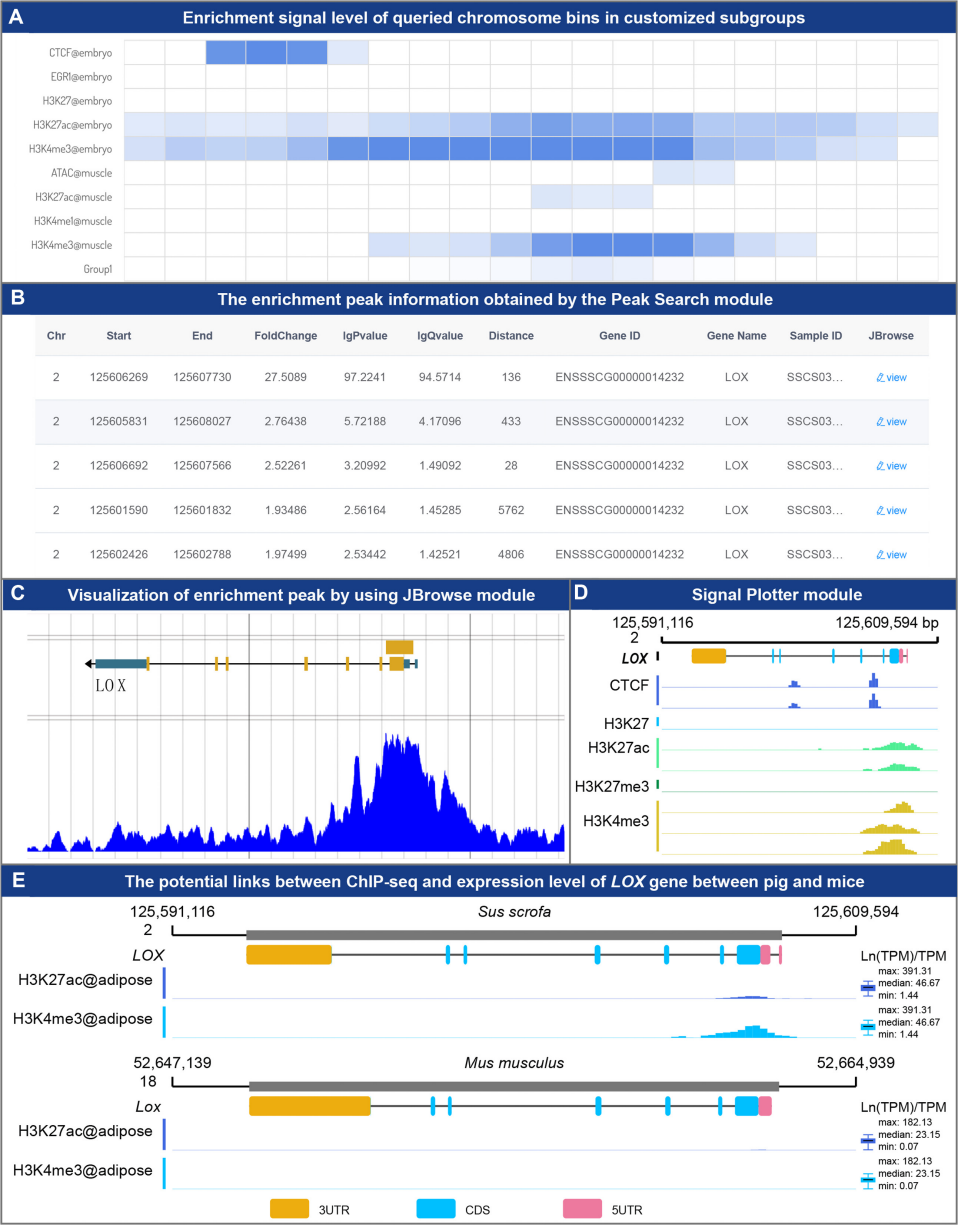
The main functions and usage of the Epigenome section. (**A**) Enrichment signal level of queried chromosome bins in customized subgroups. (**B**) The enrichment peak information obtained by the Peak Search module. Users can click the view button to display the peak on JBrowse. (**C**) Visualization of user-specified enrichment peak by using the JBrowse module. Users can also add other tracks to compare with this peak. (**D**) Example in which the Signal Plotter module was used to visualize the enrichment signals of CTCT, H3K27, H3K27ac, H3K27me3 and H3K4me3 in the *LOX* gene region in embryo tissue. This module can display the enrichment signals of multiple samples. (**E**) Example in which the Signal Comparison module was used to reveal potential links between ChIP-seq, ATAC-seq, and expression level of *LOX* gene between pig and mice.

The Epigenome section contains five modules: Signal View, Peak Search, Signal Plotter, Signal Comparison and Data Matrix. Using the Signal View module, the enrichment signals of specified regions in different targets and tissues can be obtained by searching gene ID or genomic region. The Signal View module provides two modes, selection by target/tissue and custom grouping, which helps users construct any number of subgroups (Supplementary Figure S4), and the retrieved results will be exhibited in the heatmap (Figure 5A). To make it easier for users to customize subgroups with sample information, this section also provides the Data Matrix module to help users view the epigenomics data in IAnimal more intuitively (Supplementary Figure S5). In addition to enrichment signals, users can also view enrichment peaks and their statistical information in a specified genome region through the Peak Search module (Figure 5B). By clicking the link in the results, the genome coverage of the sample corresponding to the peak can be conveniently viewed in the JBrowse module (Figure 5C). In addition, although the coverage of a specified region for the samples of different targets and tissue near a specified gene can be viewed through the JBrowse track file provided by IAnimal, it is difficult to merge and to visualize a large number of samples in JBrowse. We implemented the Signal Plotter module by using IAnimal's flexible data API, which can merge samples in the specified group and return a publication-level vector diagram (Figure 5D) and users can specify one or more groups for visualization. IAnimal also provides a Signal Comparison module to easily reveal potential links between ChIP-seq, ATAC-seq and expression levels of given genes across species. Using this module, users can easily compare the signals and expression levels of a given gene between two species (Figure 5E).

### Literature section

**Figure 6.**
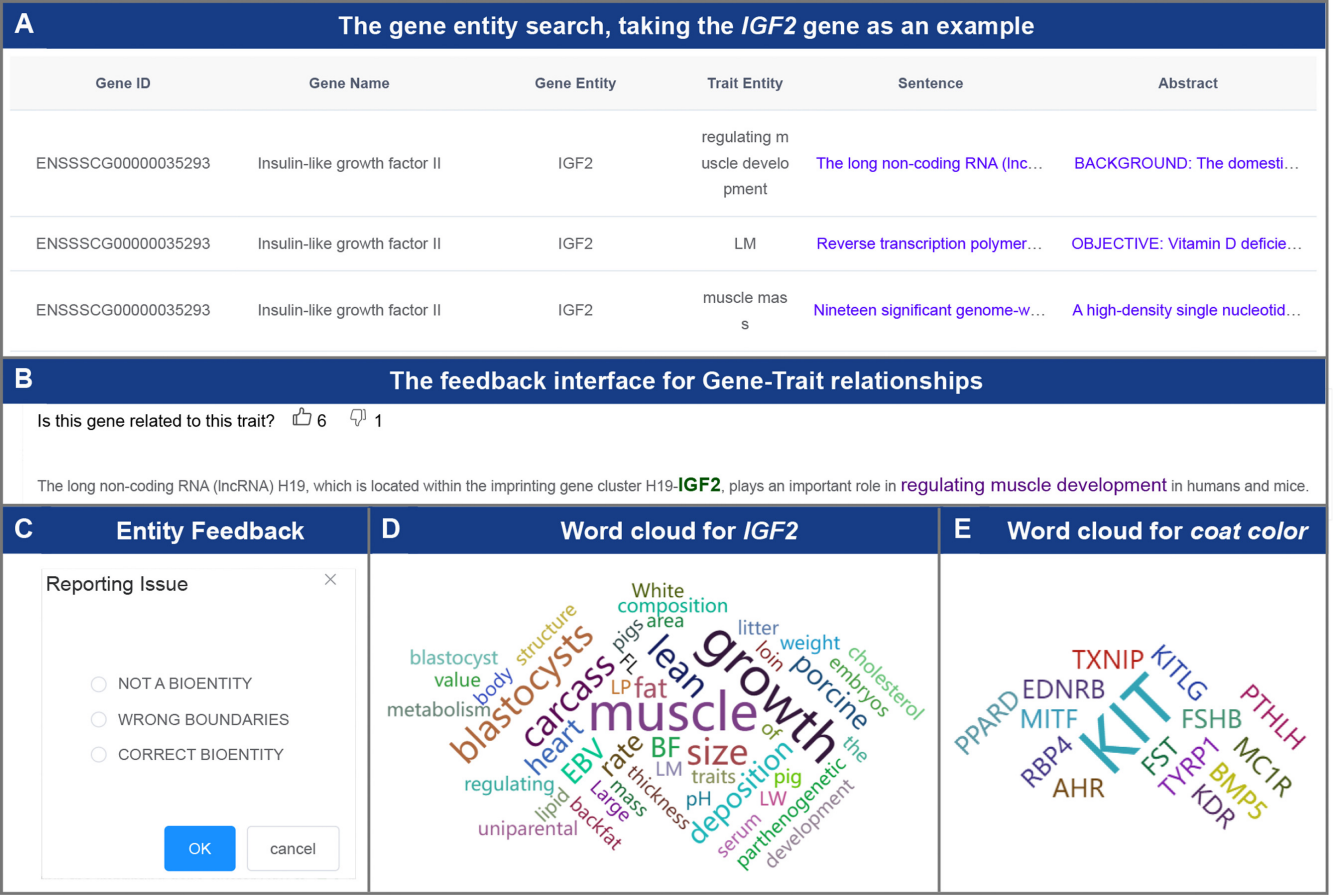
The main functions and usage of the Literature section. (**A**) The results of the Entity Search module, using the *IGF2* gene as an example. The results contain genes and phenotype entities, and users can click the corresponding sentence or abstract to view the detailed information. (**B**) The feedback interface for Gene-Trait relationships. This allows users to give feedback on the reliability of relationships between genes and traits. (**C**) The feedback interface for entity recognition. Users can feed back the accuracy of entity recognition, which will assist the continuous optimization of the named entity recognition model. (**D**) The word cloud image generated by the Entity Cloud module, using the *IGF2* gene as an example. From the word cloud image, users can infer that *IGF2* may be related to muscle growth. (**E**) The word cloud image generated by the Entity Cloud module, using coat color trait as an example. From the word cloud image, users can infer that the *KIT* gene plays an important role in regulating this trait.

The Literature section includes the two modules: Entity Search and Entity Cloud. Users can retrieve gene or phenotype entities in the Entity Search module, which will return detailed descriptions and abstract information related to the corresponding entities (Figure 6A); then, users can comprehensively evaluate the potential functions of the specified genes and the potential regulatory gene sets of the specified traits. Because these entities are derived from machine learning models, false positives cannot be avoided completely. This module also provides a convenient feedback function to optimize the model continuously to improve the accuracy of entity recognition (Figure 6B, C). To facilitate intuitive exploration of gene functions and trait-related genes, this section also provides the Entity Cloud module, which displays the search results as a word cloud so that the information provided by the literature is clear at a glance (Figure 6D, E).

### Tools section

The Tools section contains five modules: JBrowse, BLAST, Primer, Enrichment and Data API. The JBrowse module enables users to visualize genomes, genes, variants, ChIP-Seq, and ATAC-Seq signals for 21 species at the genome-wide level and to derive high-quality vectorgraphs. Through the BLAST module, users can align the specified nucleic acid sequences or protein sequences to genomics, CDS, cDNA, ncRNA, and protein sequences of specified species online, which is convenient for sequence function research. With the Primer module, users can design primers for downstream experimental validation. The Enrichment module can be used to perform GO and KEGG functional enrichment analysis on a specified gene set. The Data API module is the basis for the efficient use of multi-omics big data in the IAnimal knowledgebase. The API interface helps users acquire multi-omics data more flexibly for personalized analysis and visualization; it provides 12 types of interfaces, namely, Species, Expression, Genes, Variation, Epigenome, Literature, Homology, Gene NetWork, Annotation, Gene Family, Statistics and Plotter. By referring to the demo, users can obtain the data of interest. However, in contrast to the simpler modules in the Tools section, the use of the Data API module requires certain programming skills and experience. In the future, easier, faster, and more convenient online tools will be generated for these interfaces to meet the requirements of users worldwide.

### Taxonomy, download, and help modules

The Taxonomy module mainly introduces the species in this study and their omics data, which is convenient for users to obtain the basic information for each species. The Download module was designed to obtain genome-related files and various omics information used in the knowledgebase for local excavation. The Help module contains the introduction, user manual, FAQs, and update&news for IAnimal, in which users can obtain detailed information about the database and provide valuable comments and constructive suggestions.

## SUMMARY AND FUTURE DIRECTIONS

With the continuous development of experimental techniques and sequencing technology, multi-omics data have exhibited hyper-exponential growth. However, it is still a major challenge to unite and utilize these very large data sets to systematically explore the genetic mechanisms that underlie the formation of a trait, especially in the domain of animal studies. Most existing animal databases, such as AnimalTFDB (48), AnimalQTLdb (7), Animal-ImputeDB (49) and Animal-eRNAdb (8), focus mainly on a single type of omics data. In this area of research, IAnimal is currently the most comprehensive multi-omics database, covering the largest number of animal species. At present, IAnimal includes 61 191 individual level omics data (e.g. WGS, RNA-Seq, ChIP-Seq and ATAC-Seq) and genome annotation information for 21 animal species, and its scale of clean data is 846.46 TB. IAnimal includes a novel deep learning model developed based on the BioBERT and AutoNER algorithms. This model mines the relationship between ‘gene’ and ‘trait’ by using 2 794 237 abstracts to learn the regulation pattern of different omics layers and effects of genes on traits.

By means of a user-friendly web interface, IAnimal enables users to easily query, mine, and visualize the features of genes in various omics, such as gene expression profiles in different tissues, gene networks among genes, genotyping results of variant sites, and enrichment signals around genes for different transcription factors or histones. By aid of flexible data APIs and abundant functional modules within IAnimal, users can utilize cross-species multi-omics information to mine for gene functions. With the explosive increase in the scale of multi-omics data for animals and the rapid development of deep learning frameworks such as Transformer, developing more intelligent integrated multi-omics analysis methods to interpret the relationships between genes and traits will be a direction for future work.

It should be noted that IAnimal focuses mainly on WGS, RNA-Seq, ChIP-seq, ATAC-Seq and literature data. In the future, with the increasing data volume of high-throughput/resolution chromosome conformation capture (Hi-C), whole genome bisulfite sequencing (WGBS), and other omics data types, we will continue to expand omics data and enrich IAnimal with new types of omics data. In addition, although flexible data APIs in IAnimal enable personalized data analysis, modules to facilitate downstream data analysis and visualization based on these APIs still need to be enriched. Overall, IAnimal will be committed to providing comprehensive, structured multi-omics data for a wide range of animal species as well as relevant, intelligent integration analysis algorithms and corresponding mining and visualization tools. IAnimal is a valuable resource for producing unprecedented knowledge to fill the gap between genomes and phenomes.

## DATA AVAILABILITY

IAnimal is freely available to the public at https://ianimal.pro/.

## Supplementary Material

gkac936_Supplemental_File

## References

[1] Subramanian I. , Verma S., Kumar S., Jere A., Anamika K. Multi-omics data integration, interpretation, and its application. Bioinform Biol. Insights. 2020; 14:1177932219899051. 32076369 10.1177/1177932219899051 PMC7003173

[2] Luo Y. , Hitz B.C., Gabdank I., Hilton J.A., Kagda M.S., Lam B., Myers Z., Sud P., Jou J., Lin K. et al. New developments on the encyclopedia of DNA elements (ENCODE) data portal. Nucleic Acids Res. 2020; 48:D882–D889. 31713622 10.1093/nar/gkz1062 PMC7061942

[3] FAANG Consortium Giuffra E. , Tuggle C.K. Functional annotation of animal genomes (FAANG): current achievements and roadmap. Annu. Rev. Anim. Biosci. 2019; 7:65–88. 30427726 10.1146/annurev-animal-020518-114913

[4] Fu Y. , Fan P., Wang L., Shu Z., Zhu S., Feng S., Li X., Qiu X., Zhao S., Liu X. Improvement, identification, and target prediction for miRNAs in the porcine genome by using massive, public high-throughput sequencing data. J. Anim. Sci. 2021; 99:skab018. 33493272 10.1093/jas/skab018 PMC7885162

[5] Li C. , Tian D., Tang B., Liu X., Teng X., Zhao W., Zhang Z., Song S. Genome variation map: a worldwide collection of genome variations across multiple species. Nucleic Acids Res. 2021; 49:D1186–D1191. 33170268 10.1093/nar/gkaa1005 PMC7778933

[6] Fu W. , Wang R., Nanaei H.A., Wang J., Hu D., Jiang Y. RGD v2.0: a major update of the ruminant functional and evolutionary genomics database. Nucleic Acids Res. 2022; 50:D1091–D1099. 34643708 10.1093/nar/gkab887 PMC8728256

[7] Hu Z.L. , Park C.A., Reecy J.M. Bringing the animal QTLdb and CorrDB into the future: meeting new challenges and providing updated services. Nucleic Acids Res. 2022; 50:D956–D961. 34850103 10.1093/nar/gkab1116 PMC8728226

[8] Jin W. , Jiang G., Yang Y., Yang J., Yang W., Wang D., Niu X., Zhong R., Zhang Z., Gong J. Animal-eRNAdb: a comprehensive animal enhancer RNA database. Nucleic Acids Res. 2022; 50:D46–D53. 34551433 10.1093/nar/gkab832 PMC8728245

[9] Kang M. , Ko E., Mersha T.B. A roadmap for multi-omics data integration using deep learning. Brief Bioinform. 2022; 23:bbab454. 34791014 10.1093/bib/bbab454 PMC8769688

[10] Fu Y. , Xu J., Tang Z., Wang L., Yin D., Fan Y., Zhang D., Deng F., Zhang Y., Zhang H. et al. A gene prioritization method based on a swine multi-omics knowledgebase and a deep learning model. Commun. Biol. 2020; 3:502. 32913254 10.1038/s42003-020-01233-4 PMC7483748

[11] Cunningham F. , Allen J.E., Allen J., Alvarez-Jarreta J., Amode M.R., Armean I.M., Austine-Orimoloye O., Azov A.G., Barnes I., Bennett R. et al. Ensembl 2022. Nucleic Acids Res. 2022; 50:D988–D995. 34791404 10.1093/nar/gkab1049 PMC8728283

[12] Katz K. , Shutov O., Lapoint R., Kimelman M., Brister J.R., O'Sullivan C The sequence read archive: a decade more of explosive growth. Nucleic Acids Res. 2022; 50:D387–D390. 34850094 10.1093/nar/gkab1053 PMC8728234

[13] Cantelli G. , Bateman A., Brooksbank C., Petrov A.I., Malik-Sheriff R.S., Ide-Smith M., Hermjakob H., Flicek P., Apweiler R., Birney E. et al. The european bioinformatics institute (EMBL-EBI) in 2021. Nucleic Acids Res. 2022; 50:D11–D19. 34850134 10.1093/nar/gkab1127 PMC8690175

[14] Sayers E.W. , Bolton E.E., Brister J.R., Canese K., Chan J., Comeau D.C., Connor R., Funk K., Kelly C., Kim S. et al. Database resources of the national center for biotechnology information. Nucleic Acids Res. 2022; 50:D20–D26. 34850941 10.1093/nar/gkab1112 PMC8728269

[15] Di Tommaso P. , Chatzou M., Floden E.W., Barja P.P., Palumbo E., Notredame C. Nextflow enables reproducible computational workflows. Nat. Biotechnol. 2017; 35:316–319. 28398311 10.1038/nbt.3820

[16] Tkachenko M. , Malyuk M., Shevchenko N., Holmanyuk A., Liubimov N. Label studio: data labeling software, 2020-2022. 2022; *Open source software available from**GitHub*.

[17] Quevillon E. , Silventoinen V., Pillai S., Harte N., Mulder N., Apweiler R., Lopez R. InterProScan: protein domains identifier. Nucleic Acids Res. 2005; 33:W116–W120. 15980438 10.1093/nar/gki442 PMC1160203

[18] Aramaki T. , Blanc-Mathieu R., Endo H., Ohkubo K., Kanehisa M., Goto S., Ogata H. KofamKOALA: KEGG ortholog assignment based on profile HMM and adaptive score threshold. Bioinformatics. 2020; 36:2251–2252. 31742321 10.1093/bioinformatics/btz859 PMC7141845

[19] Boutet E. , Lieberherr D., Tognolli M., Schneider M., Bansal P., Bridge A.J., Poux S., Bougueleret L., Xenarios I. UniProtKB/Swiss-Prot, the manually annotated section of the uniprot knowledgebase: how to use the entry view. Methods Mol. Biol. 2016; 1374:23–54. 26519399 10.1007/978-1-4939-3167-5_2

[20] Kanehisa M. , Goto S. KEGG: kyoto encyclopedia of genes and genomes. Nucleic Acids Res. 2000; 28:27–30. 10592173 10.1093/nar/28.1.27 PMC102409

[21] Gene Ontology, C. The gene ontology resource: enriching a GOld mine. Nucleic Acids Res. 2021; 49:D325–D334. 33290552 10.1093/nar/gkaa1113 PMC7779012

[22] Mistry J. , Chuguransky S., Williams L., Qureshi M., Salazar G.A., Sonnhammer E.L.L., Tosatto S.C.E., Paladin L., Raj S., Richardson L.J. et al. Pfam: the protein families database in 2021. Nucleic Acids Res. 2021; 49:D412–D419. 33125078 10.1093/nar/gkaa913 PMC7779014

[23] Blum M. , Chang H.Y., Chuguransky S., Grego T., Kandasaamy S., Mitchell A., Nuka G., Paysan-Lafosse T., Qureshi M., Raj S. et al. The interpro protein families and domains database: 20 years on. Nucleic Acids Res. 2021; 49:D344–D354. 33156333 10.1093/nar/gkaa977 PMC7778928

[24] Tatusov R.L. , Fedorova N.D., Jackson J.D., Jacobs A.R., Kiryutin B., Koonin E.V., Krylov D.M., Mazumder R., Mekhedov S.L., Nikolskaya A.N. et al. The COG database: an updated version includes eukaryotes. BMC Bioinf. 2003; 4:41. 10.1186/1471-2105-4-41 PMC222959 12969510

[25] Emms D.M. , Kelly S. OrthoFinder: phylogenetic orthology inference for comparative genomics. Genome Biol. 2019; 20:238. 31727128 10.1186/s13059-019-1832-y PMC6857279

[26] Liu F. , Li Y., Yu H., Zhang L., Hu J., Bao Z., Wang S. MolluscDB: an integrated functional and evolutionary genomics database for the hyper-diverse animal phylum mollusca. Nucleic Acids Res. 2021; 49:D988–D997. 33219670 10.1093/nar/gkaa918 PMC7779068

[27] Kodama Y. , Shumway M., Leinonen R. International Nucleotide Sequence Database, C. The sequence read archive: explosive growth of sequencing data. Nucleic Acids Res. 2012; 40:D54–D56. 22009675 10.1093/nar/gkr854 PMC3245110

[28] Chen S. , Zhou Y., Chen Y., Gu J. fastp: an ultra-fast all-in-one FASTQ preprocessor. Bioinformatics. 2018; 34:i884–i890. 30423086 10.1093/bioinformatics/bty560 PMC6129281

[29] Li H. , Durbin R. Fast and accurate short read alignment with burrows-wheeler transform. Bioinformatics. 2009; 25:1754–1760. 19451168 10.1093/bioinformatics/btp324 PMC2705234

[30] Freed D. , Aldana R., Weber J.A., Edwards J.S. The sentieon genomics tools—a fast and accurate solution to variant calling from next-generation sequence data. 2017; bioRxiv doi:12 May 2017, preprint: not peer reviewed 10.1101/115717.

[31] McKenna A. , Hanna M., Banks E., Sivachenko A., Cibulskis K., Kernytsky A., Garimella K., Altshuler D., Gabriel S., Daly M. et al. The genome analysis toolkit: a mapreduce framework for analyzing next-generation DNA sequencing data. Genome Res. 2010; 20:1297–1303. 20644199 10.1101/gr.107524.110 PMC2928508

[32] Wang K. , Li M., Hakonarson H. ANNOVAR: functional annotation of genetic variants from high-throughput sequencing data. Nucleic Acids Res. 2010; 38:e164. 20601685 10.1093/nar/gkq603 PMC2938201

[33] Sherry S.T. , Ward M.H., Kholodov M., Baker J., Phan L., Smigielski E.M., Sirotkin K. dbSNP: the NCBI database of genetic variation. Nucleic Acids Res. 2001; 29:308–311. 11125122 10.1093/nar/29.1.308 PMC29783

[34] Price M.N. , Dehal P.S., Arkin A.P. FastTree 2–approximately maximum-likelihood trees for large alignments. PLoS One. 2010; 5:e9490. 20224823 10.1371/journal.pone.0009490 PMC2835736

[35] Kim D. , Paggi J.M., Park C., Bennett C., Salzberg S.L. Graph-based genome alignment and genotyping with HISAT2 and HISAT-genotype. Nat. Biotechnol. 2019; 37:907–915. 31375807 10.1038/s41587-019-0201-4 PMC7605509

[36] Pertea M. , Pertea G.M., Antonescu C.M., Chang T.C., Mendell J.T., Salzberg S.L. StringTie enables improved reconstruction of a transcriptome from RNA-seq reads. Nat. Biotechnol. 2015; 33:290–295. 25690850 10.1038/nbt.3122 PMC4643835

[37] Zhang H. , Song L., Wang X., Cheng H., Wang C., Meyer C.A., Liu T., Tang M., Aluru S., Yue F. et al. Fast alignment and preprocessing of chromatin profiles with chromap. Nat. Commun. 2021; 12:6566. 34772935 10.1038/s41467-021-26865-w PMC8589834

[38] Liu T. Use model-based analysis of chip-Seq (MACS) to analyze short reads generated by sequencing protein-DNA interactions in embryonic stem cells. Methods Mol. Biol. 2014; 1150:81–95. 24743991 10.1007/978-1-4939-0512-6_4

[39] Kharchenko P.V. , Tolstorukov M.Y., Park P.J. Design and analysis of chip-seq experiments for DNA-binding proteins. Nat. Biotechnol. 2008; 26:1351–1359. 19029915 10.1038/nbt.1508 PMC2597701

[40] Lee B.T. , Barber G.P., Benet-Pages A., Casper J., Clawson H., Diekhans M., Fischer C., Gonzalez J.N., Hinrichs A.S., Lee C.M. et al. The UCSC genome browser database: 2022 update. Nucleic Acids Res. 2022; 50:D1115–D1122. 34718705 10.1093/nar/gkab959 PMC8728131

[41] Lee J. , Yoon W., Kim S., Kim D., Kim S., So C.H., Kang J. BioBERT: a pre-trained biomedical language representation model for biomedical text mining. Bioinformatics. 2020; 36:1234–1240. 31501885 10.1093/bioinformatics/btz682 PMC7703786

[42] Shang J. , Liu L., Ren X., Gu X., Ren T., Han J. Learning named entity tagger using domain-specific dictionary. Proceedings of the 2018 Conference on Empirical Methods in Natural Language Processing. 2018; Brussels, Belgium Association for Computational Linguistics 2054–2064.

[43] Smith C.L. , Eppig J.T. Expanding the mammalian phenotype ontology to support automated exchange of high throughput mouse phenotyping data generated by large-scale mouse knockout screens. J. Biomed. Semantics. 2015; 6:11. 25825651 10.1186/s13326-015-0009-1 PMC4378007

[44] Park C.A. , Bello S.M., Smith C.L., Hu Z.L., Munzenmaier D.H., Nigam R., Smith J.R., Shimoyama M., Eppig J.T., Reecy J.M. The vertebrate trait ontology: a controlled vocabulary for the annotation of trait data across species. J. Biomed. Semantics. 2013; 4:13. 23937709 10.1186/2041-1480-4-13 PMC3851175

[45] Buels R. , Yao E., Diesh C.M., Hayes R.D., Munoz-Torres M., Helt G., Goodstein D.M., Elsik C.G., Lewis S.E., Stein L. et al. JBrowse: a dynamic web platform for genome visualization and analysis. Genome Biol. 2016; 17:66. 27072794 10.1186/s13059-016-0924-1 PMC4830012

[46] Priyam A. , Woodcroft B.J., Rai V., Moghul I., Munagala A., Ter F., Chowdhary H., Pieniak I., Maynard L.J., Gibbins M.A. et al. Sequenceserver: a modern graphical user interface for custom BLAST databases. Mol. Biol. Evol. 2019; 36:2922–2924. 31411700 10.1093/molbev/msz185 PMC6878946

[47] Untergasser A. , Cutcutache I., Koressaar T., Ye J., Faircloth B.C., Remm M., Rozen S.G. Primer3–new capabilities and interfaces. Nucleic Acids Res. 2012; 40:e115. 22730293 10.1093/nar/gks596 PMC3424584

[48] Hu H. , Miao Y.R., Jia L.H., Yu Q.Y., Zhang Q., Guo A.Y. AnimalTFDB 3.0: a comprehensive resource for annotation and prediction of animal transcription factors. Nucleic Acids Res. 2019; 47:D33–D38. 30204897 10.1093/nar/gky822 PMC6323978

[49] Yang W. , Yang Y., Zhao C., Yang K., Wang D., Yang J., Niu X., Gong J. Animal-ImputeDB: a comprehensive database with multiple animal reference panels for genotype imputation. Nucleic Acids Res. 2020; 48:D659–D667. 31584087 10.1093/nar/gkz854 PMC6943029

