# Supplementary material for: IAnimal: a cross-species omics knowledgebase for animals
Source: Nucleic Acids Res. 2022 Oct 27;51(D1):D1312–24. doi: 10.1093/nar/gkac936 (PMC9825575; doi:10.1093/nar/gkac936)
Supplement: gkac936_Supplemental_File [file gkac936_supplemental_file.pdf]

Supplementary materials to

IAnimal: A cross-species omics knowledgebase for animals

Yuhua Fu[1,2], Hong Liu[1], Jingwen Dou[1], Yue Wang[1], Yong Liao[1], Xin Huang[1], Zhenshuang Tang[1], JingYa Xu[1], Dong Yin[1], Shilin Zhu[1], Yangfan Liu[1], Xiong Shen[1], Hengyi Liu[1], Jiaqi Liu[1], Xin Yang[1], Yi Zhang[4], Yue Xiang[1], Jingjin Li[1], Zhuqing Zheng[1], Yunxia Zhao[1,2], Yunlong Ma[1,2], Haiyan Wang[2], Xiaoyong Du[2], Shengsong Xie[1,2], Xuewen Xu[1,2], Haohao Zhang[4], Lilin Yin[1,2], Mengjin Zhu[1,2], Mei Yu[1,2], Xinyun Li[1,2], Xiaolei Liu[1,2,3,*], and Shuhong Zhao[1,2,3,*]

[1] Key Laboratory of Agricultural Animal Genetics, Breeding and Reproduction, Ministry of Education, Key Laboratory of Swine Genetics and Breeding, Ministry of Agriculture, College of Animal Science and Technology, Huazhong Agricultural University, Wuhan, Hubei, 430070, PR China;

[2] Frontiers Science Center for Animal Breeding and Sustainable Production, Wuhan, Hubei, 430070, PR China;

[3] Hubei Hongshan Laboratory, Wuhan, Hubei, 430070, PR China;

[4] School of Computer Science and Technology, Wuhan University of Technology, Wuhan, Hubei, 430070, PR China.

* To whom correspondence should be addressed. Tel: +86 (027) 87387480; Email: shzhao@mail.hzau.edu.cn

Correspondence should also be addressed to Xiaolei Liu. Email: xiaoleiliu@mail.hzau.edu.cn

Supplementary Results

Construction of named entity recognition model

A total of 1,760 abstracts were prepared for training the named entity recognition model, which consisted of 25,785 gene entities and 18,328 phenotype entities. In view of the excellent performance of “BioBERT,” which is a pretrained biomedical language representation model for biomedical text mining, our study fine-tuned the BioBERT model using this training set, and found that the accuracy, precision, recall, and F1-Measure of the BioBERT-based model were 95.16%, 77.09%, 84.69%, and 80.71%, respectively (**Table R1**). To further identify more entities, we adopted the dictionary-based named entity recognition algorithm “AutoNER”. After applying this algorithm, we observed that the number of recognized entities increased from 8,800 to 10,493 (19.24%). Finally, we mapped each identified entity to a unified gene ID or a professional trait ontology in an expert-defined entity dictionary for the convenience of users. The model precision increased from 65.57% to 78.39%, which indicates that the gene and phenotype entities provided by the Entity-related modules have a low false positive rate.

Table R1. The performance of the named entity recognition models.

Models	Accuracy	Precision	Recall	F1-Measure	#Gene	#Phenotype
BioBERT	95.16%	77.09%	84.69%	80.71%	5,253	3,547
BioBERT+AutoNER	94.54%	65.57%	78.25%	71.35%	6,062	4,431
BioBERT+AutoNER-mapping	89.95%	78.39%	32.19%	45.64%	1,640	1,695

Supplementary Figures

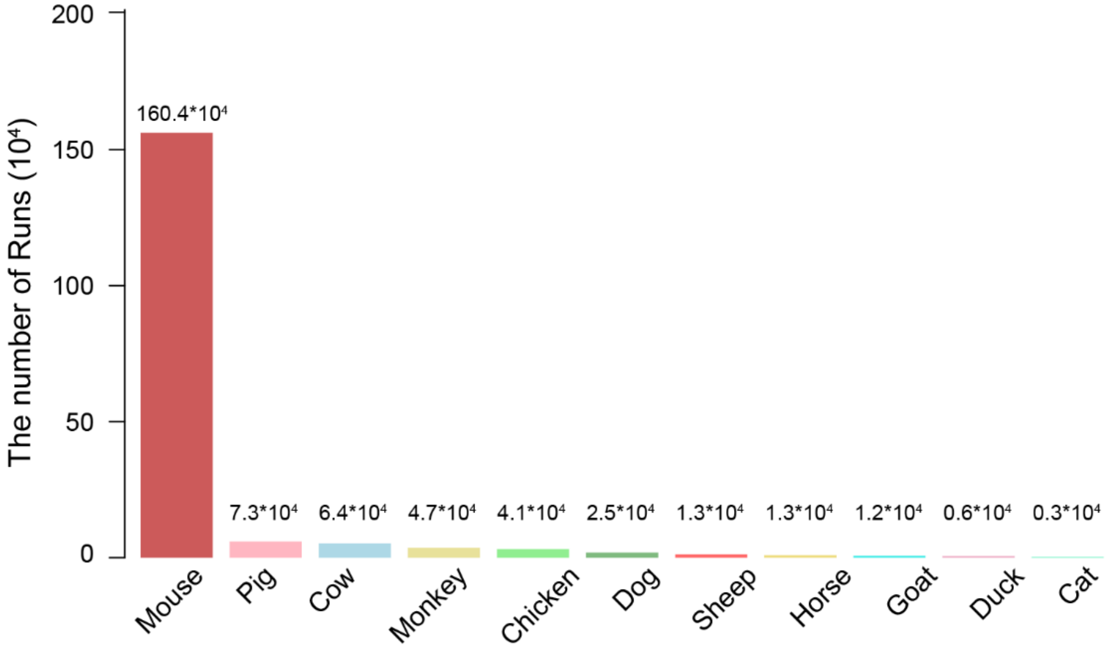

Supplementary Figure 1. Comparison of published Next-Generation Sequencing (NGS) data volumes for common model/non-model animals. The published multi-omics data for mice are typically 20–500 times greater in volume than data for other animal species. The Y-axis represents the number of SRA Runs, which is simply a manifestation of data file(s) that were derived from sequencing a library described by the associated experiment. The X-axis represents the varied animal species.

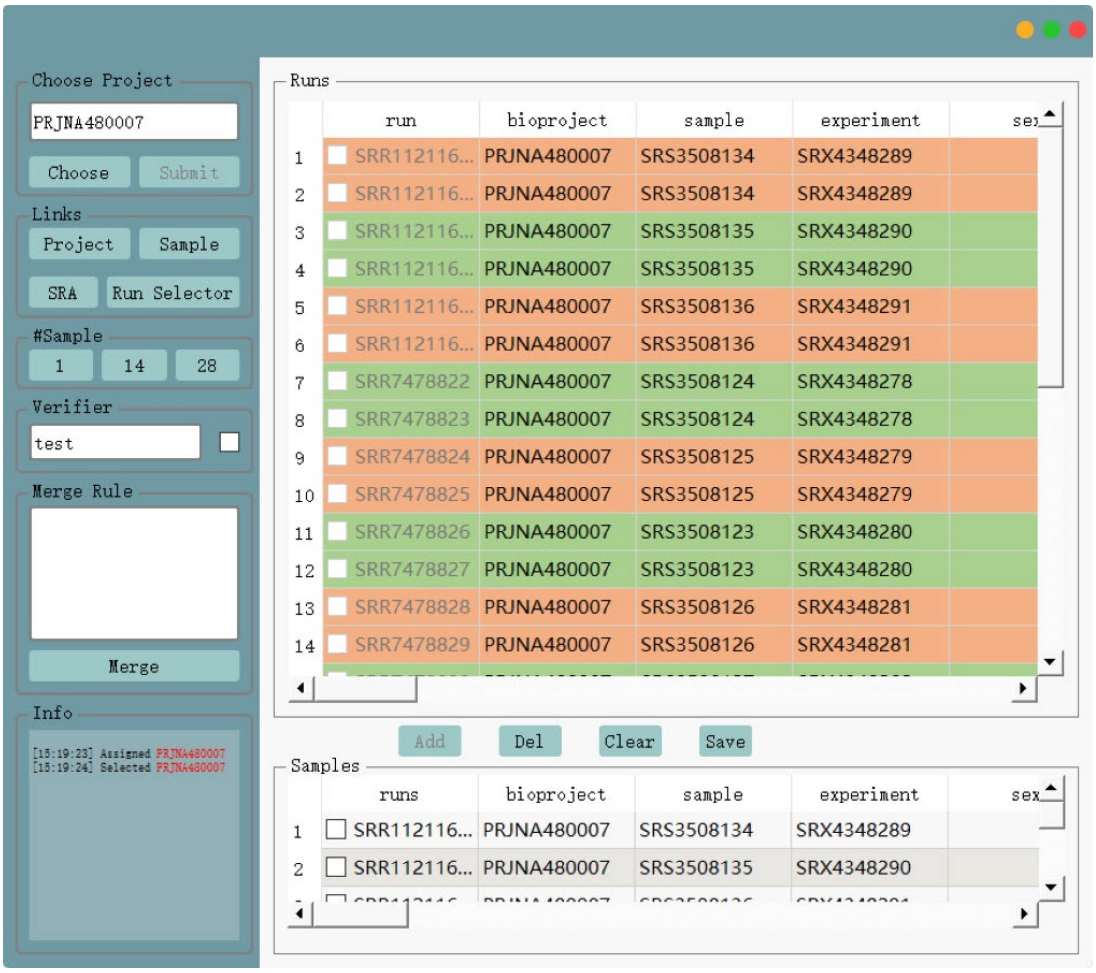

Supplementary Figure 2. The interface of the NGS data cleaning program. The program assigns cleaning tasks and provides auxiliary functions to help volunteers clean NGS data. It also stores the results in an online database and completes error correction by comparing the cleaning results of multiple volunteers.

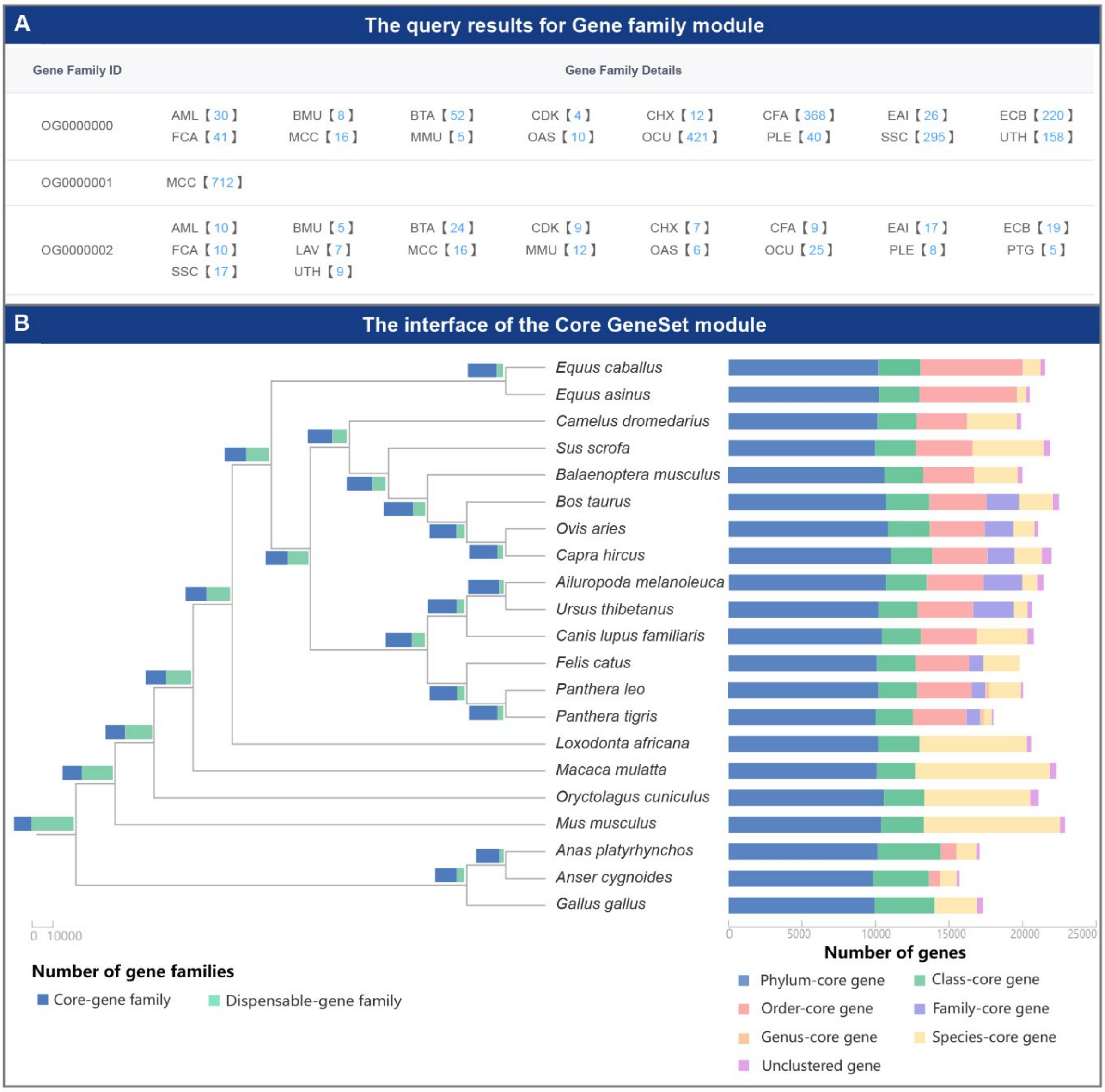

Supplementary Figure 3. The interface of the Gene family and Core GeneSet module. **(A)** The query results for the Gene family module. Each cluster is divided according to species, and users can choose species of interest to obtain more detailed information. **(B)** The interface of the Core GeneSet module. The core and dispensable gene families were counted in different evolutionary branches, and the core genes of different species were identified simultaneously at different phylogenetic levels, which included the phylum, class, order, family, genus, and species. Users can download the data of interest by clicking on the corresponding bar.

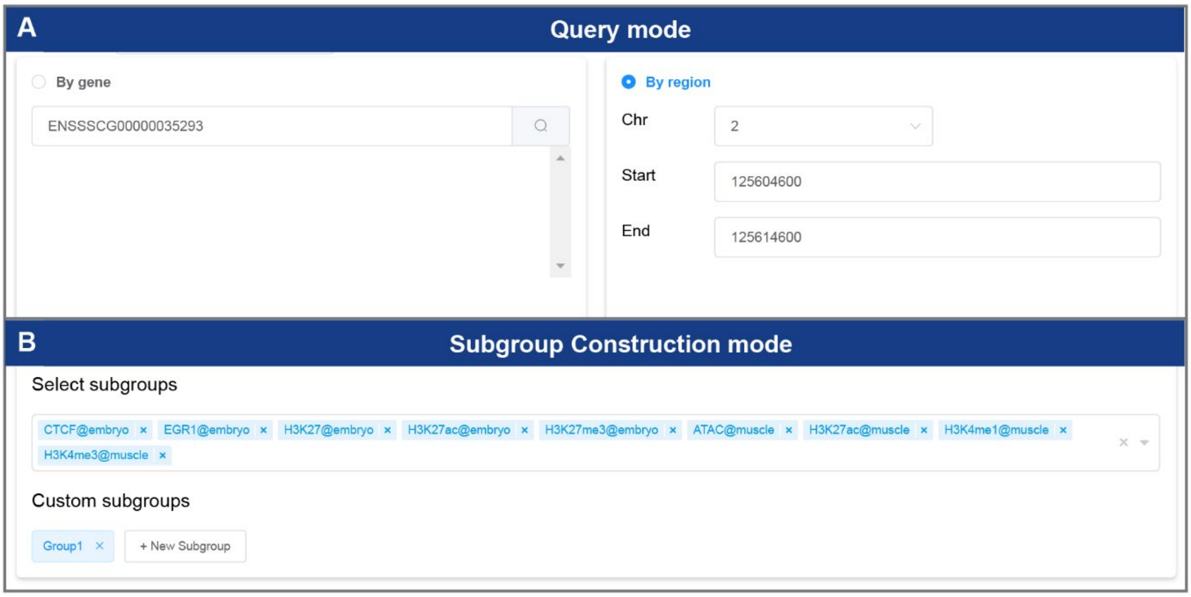

Supplementary Figure 4. Query mode and subgroup construction mode in the Signal View module. (A) Two different search modes. Users can select an appropriate mode to obtain the genomic regions of interest. **(B)** Two methods of constructing subgroups. Using the "Custom mode" or "Select mode" by target/tissue can help users quickly construct one or more subgroups.

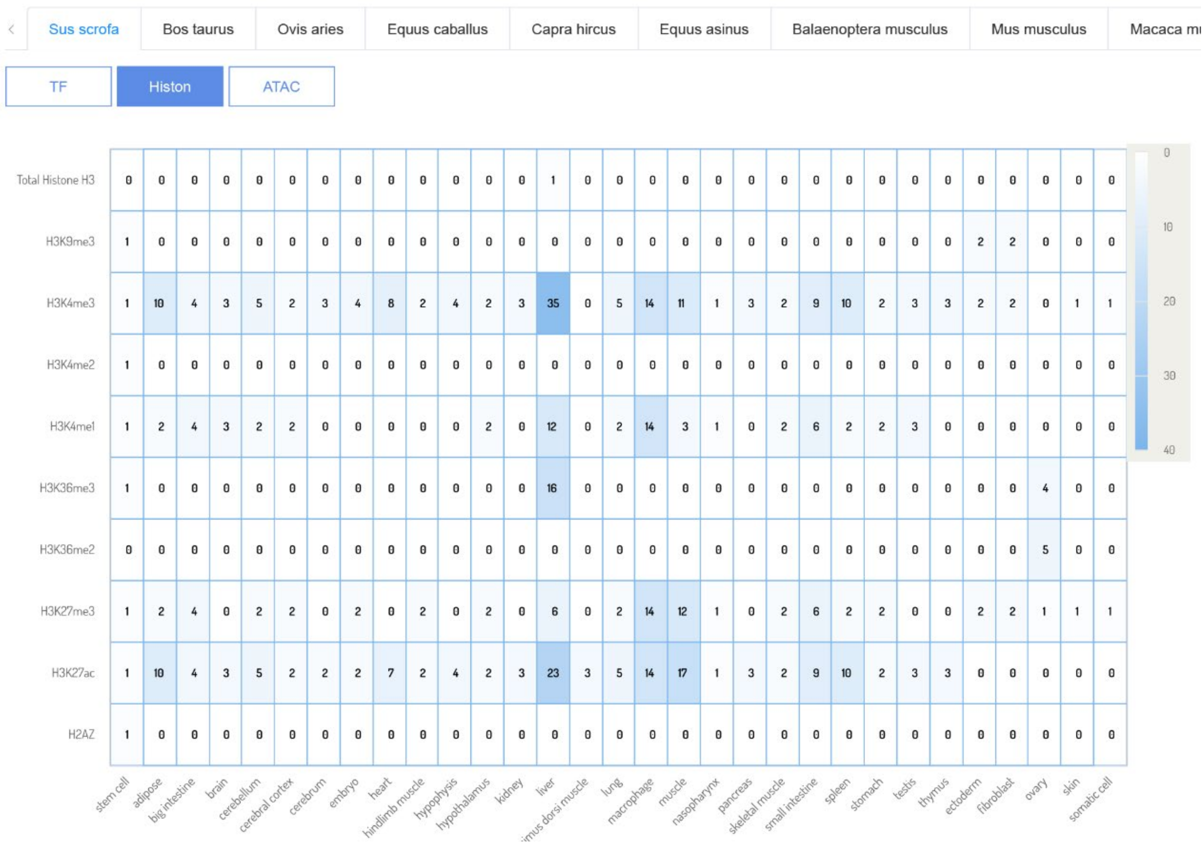

Supplementary Figure 5. The interface of the Data Matrix module. Users can intuitively view the number, target, and tissue of the epigenomics data of a specified species and select appropriate samples for downstream analyses in modules like Signal View, Peak Search, and Signal Plotter.

Supplementary Tables

Supplementary Table 1. Gene function annotation of 21 species in different databases.

Species	Genes	Swiss-Prot	KEGG	GO	Pfam	InterPro	KOG
Ailuropoda melanoleuca	24,463	20,295	15,775	14,847	18,915	19,596	18,246
Anas platyrhynchos	18,491	15,871	12,099	11,811	15,235	15,779	14,433
Anser cygnoides	19,449	14,734	11,593	11,032	14,010	14,524	13,405
Balaenoptera musculus	22,592	19,038	14,746	13,724	17,599	18,245	16,980
Bos taurus	27,608	21,224	16,772	15,410	19,968	20,612	19,096
Camelus dromedarius	22,445	18,878	14,801	13,762	17,604	18,195	16,862
Canis lupus familiaris	30,952	19,760	15,442	14,255	18,483	19,104	17,837
Capra hircus	27,272	20,923	16,621	15,502	19,620	20,291	18,872
Equus asinus	22,929	19,425	15,053	14,114	18,043	18,685	17,453
Equus caballus	30,372	20,367	16,038	14,696	19,292	19,851	18,334
Felis catus	29,551	19,019	15,183	13,989	17,858	18,452	17,053
Gallus gallus	24,357	15,920	12,269	11,799	15,252	15,842	14,465
Loxodonta africana	23,246	19,793	15,739	14,685	18,491	19,081	17,938
Macaca mulatta	35,433	21,036	15,102	13,646	18,250	18,802	19,161
Mus musculus	55,417	21,373	17,288	15,889	20,606	21,156	18,964
Oryctolagus cuniculus	29,588	19,997	14,793	14,064	18,576	19,250	18,082
Ovis aries	26,479	19,959	15,808	14,758	18,724	19,338	17,955
Panthera leo	22,744	19,054	15,091	13,893	17,831	18,438	17,056
Panthera tigris	22,120	17,231	13,084	12,454	15,867	16,491	15,450
Sus scrofa	31,909	20,041	15,562	14,436	18,871	19,453	18,202
Ursus thibetanus	23,211	19,560	15,553	14,234	18,380	18,942	17,525
**All**	**570,628**	**403,498**	**314,412**	**293,000**	**377,475**	**390,127**	**363,369**

A total of 570,628 genes of 21 species were annotated in the Swiss-Prot, Kyoto Encyclopedia of Genes and Genomes (KEGG), Gene Ontology (GO), Pfam, InterPro, and KOG databases by using a unified pipeline. After this performance, the proportion of genes with the annotation information of Swiss-Prot, KEGG, GO, Pfam, InterPro, and KOG is 70.71%, 55.10%, 51.35%, 66.15%, 68.37%, and 63.68%, respectively.
